# Antiretroviral treatment use, co-morbidities and clinical outcomes among Aboriginal participants in the Australian HIV Observational Database (AHOD)

**DOI:** 10.1186/s12879-015-1051-4

**Published:** 2015-08-12

**Authors:** David J. Templeton, Stephen T. Wright, Hamish McManus, Chris Lawrence, Darren B. Russell, Matthew G. Law, Kathy Petoumenos, on behalf of the Australian HIV Observational Database

**Affiliations:** The Kirby Institute, UNSW Australia, Sydney, NSW 2052 Australia; RPA Sexual Health, Sydney Local Health District, Camperdown, NSW 2050 Australia; Central Clinical School, The University of Sydney, Sydney, NSW 2006 Australia; The George Institute for Global Health, Level 10, King George V Building, 83-117 Missenden Rd, Camperdown, NSW 2050 Australia; Cairns Sexual Health Service, PO Box 902, Cairns, QLD 4870 Australia; The University of Melbourne, VIC, Australia; James Cook University, Queensland, Australia

**Keywords:** HIV, Cohort study, Indigenous, Australia

## Abstract

**Background:**

There are few data regarding clinical care and outcomes of Indigenous Australians living with HIV and it is unknown if these differ from non-Indigenous HIV-positive Australians.

**Methods:**

AHOD commenced enrolment in 1999 and is a prospective cohort of HIV-positive participants attending HIV outpatient services throughout Australia, of which 20 (74 %) sites report Indigenous status. Data were collected up until March 2013 and compared between Indigenous and non-Indigenous participants. Person-year methods were used to compare death rates, rates of loss to follow-up and rates of laboratory testing during follow-up between Indigenous and non-Indigenous participants. Factors associated with time to first combination antiretroviral therapy (cART) regimen change were assessed using Kaplan Meier and Cox Proportional hazards methods.

**Results:**

Forty-two of 2197 (1.9 %) participants were Indigenous. Follow-up amongst Indigenous and non-Indigenous participants was 332 & 16270 person-years, respectively. HIV virological suppression was achieved in similar proportions of Indigenous and non-Indigenous participants 2 years after initiation of cART (81.0 % vs 76.5 %, *p* = 0.635). Indigenous status was not independently associated with shorter time to change from first- to second-line cART (aHR 0.95, 95 % CI 0.51-1.76,* p* = 0.957). Compared with non-Indigenous participants, Indigenous participants had significantly less frequent laboratory monitoring of CD4 count (rate:2.76 tests/year vs 2.97 tests/year, *p* = 0.025) and HIV viral load (rate:2.53 tests/year vs 2.93 tests/year,* p* < 0.001), while testing rates for lipids and blood glucose were almost half that of non-indigenous participants (rate:0.43/year vs 0.71 tests/year,* p* < 0.001). Loss to follow-up (23.8 % vs 29.8 %,* p* = 0.496) and death (2.4 % vs 7.1 %,* p* = 0.361) occurred in similar proportions of indigenous and non-Indigenous participants, respectively, although causes of death in both groups were mostly non-HIV-related.

**Conclusions:**

As far as we are aware, these are the first data comparing clinical outcomes between Indigenous and non-Indigenous HIV-positive Australians. The forty-two Indigenous participants represent over 10 % of all Indigenous Australians ever diagnosed with HIV. Although outcomes were not significantly different, Indigenous patients had lower rates of laboratory testing for HIV and lipid/glucose parameters. Given the elevated risk of cardiovascular disease in the general Indigenous community, the additional risk factor of HIV infection warrants further focus on modifiable risk factors to maximise life expectancy in this population.

## Background

The rate of HIV diagnoses among Australian Aboriginal and Torres Strait Islander (hereafter “Indigenous”) people over the past decade has been similar to that among non-Indigenous Australians [1]. However, factors such as high rates of sexually transmitted infections, social disadvantage and poor access to health services may lead to an elevated risk of HIV infection [2], as has been witnessed among Canada’s Indigenous population [3].

Australia’s Indigenous people are a heterogenous group, and the diversity of language, socio-cultural, geographic and economic factors will impact on management of Indigenous individuals living with HIV [4]. Compliance with HIV therapy is recognised as a key factor in disease progression and prognosis [5], and although challenging in any setting, it is likely to be complicated by a number of these factors in Indigenous Australians [6]. Indigenous people have poorer overall health outcomes and poor access to health services [7] which is of specific concern among those living with HIV as failure to access HIV care is also an important risk factor for HIV disease progression [5]. Such issues, along with other co-morbidities in this population, have the potential to considerably lower survival rates in Indigenous HIV-infected individuals [6].

There are few published data regarding clinical outcomes and co-morbidities of Indigenous Australians living with HIV and it is unknown if these differ from non-Indigenous HIV-positive Australians. Our aim was to describe demographic factors, clinical characteristics, and co-morbidities among Indigenous participants, and compare them with those of non-Indigenous participants enrolled in a large Australian clinical cohort of HIV-positive patients.

## Methods

The Australian HIV Observational Database (AHOD) was established in July 1999. One of the primary objectives of AHOD is to monitor trends in combination antiretroviral therapy (cART) use and HIV disease outcomes among patients with HIV in Australia; areas in which data from Indigenous individuals are lacking. At the time of the present analysis, there were 27 AHOD sites around Australia including HIV tertiary referral centres, HIV specialist general practice clinics and sexual health clinics. Consecutive clinic patients were invited to participate in AHOD in a non-random manner, thus recruitment of Indigenous patients was coincidental. Key clinical data are transferred to the co-ordinating centre, the Kirby Institute, UNSW Australia, on a 6-monthly basis. These data include patient demographics, HIV disease stage, and antiretroviral treatment history. Participant deaths are reported by study sites to The Kirby Institute AHOD team. The deceased’s attending physician enters their clinical data into a standardised coding of causes of death in HIV (CoDe) form [8] and the cause of death is then determined as being related to HIV or not. A detailed description of the AHOD cohort has previously been published [9].

### Study population

Analyses were restricted to AHOD sites that report Indigenous status, recruited to AHOD by March 2013.

### Statistical methods

Descriptive statistics were performed comparing key patient demographic and HIV related clinical information between Indigenous and non-Indigenous AHOD participants. Factors investigated include: basic demographic characteristics; HIV diagnostic characteristics: age at diagnosis, year of diagnosis, CD4 count at diagnosis & risk exposure categories; comorbidities: Hepatitis B & Hepatitis C serology results; antiretroviral treatment factors: CD4 count at commencement of cART, cART regimen prescribed, virological and immunological response to cART. Loss to follow-up (defined as not having returned to AHOD clinic in the last 12 months) and causes of death (AIDS versus non-AIDS related) were compared between Indigenous and non-Indigenous participants. Where relevant, statistical comparisons were made using chi-square tests for categorical variables, and independent t-tests or non-parametric equivalent as appropriate, for continuous variables. Person year methods were used to compare death rates, rates of loss to follow-up and of laboratory testing during follow-up between Indigenous and non-Indigenous participants. Laboratory tests included blood glucose and lipids, as well as CD4 counts and HIV viral load tests.

Factors associated with time to first cART regimen change (defined as the addition of two or more drugs to the regimen, or at least one new cART class being used) were assessed as a proxy for adherence to, and tolerability of, the initial cART regimen prescribed using Kaplan Meier and Cox Proportional hazards methods as previously described in detail [10], with *a priori* inclusion of Indigenous status in the multivariate model. Off treatment periods were included in duration to time of change. Risk factors assessed are outlined in Table 2 and the footnote to Table 2. The model was determined using backward stepwise methods. Factors in univariate analyses with *p*-value < 0.1 were assessed in the multivariate analysis. The final model contained factors with *p*-value <0.05.

Ethics approval for the AHOD study was granted by the UNSW Australia Human Research Ethics Committee, and all other relevant institutional review boards. Written informed consent was obtained from participating individuals. All study procedures were developed in accordance with the revised 1975 Helsinki Declaration.

## Results

The total number of participants recruited to AHOD by March 2013 was 3894. This analysis included six-monthly data transfers to end March 2013 for the 20 (74 %) AHOD sites which reported Indigenous status. Study participants were 2197 of 2613 (84.1 %) individuals enrolled at these 20 sites for whom Indigenous status was available. There were 42 (1.9 %) Indigenous participants in our study population.

Demographic and clinical data at the time of enrolment to AHOD are summarised in Table 1. Characteristics at enrolment were similar for participants with missing Indigenous status and those for whom Indigenous status was reported (data not shown). However, a higher proportion (8.9 %) of those with missing Indigenous status reported both male homosexual contact and injecting drug use (IDU) as potential HIV risk factors, more participants with missing Indigenous status were ARV-naïve at enrolment (41.1 %), and more of those without Indigenous status recorded were missing a CD4 count and HIV viral load measurement at enrolment (29.1 % & 33.9 %, respectively).

**Table 1:** Baseline patient characteristics by indigenous status at enrolment to the Australian HIV observational database

	Non-indigenous		Indigenous		Total	
	(*n* = 2155)		(*n* = 42)		(*N* = 2197)	
	*n*	%	*n*	%	*n*	%
Gender						
Female	100	4.6	5	11.9	105	4.8
Male	2051	95.2	36	85.7	2087	95.0
Transgender	4	0.2	1	2.4	5	0.2
Reported HIV exposure category						
Blood products	10	0.5	0	0.00	10	0.5
Heterosexual	170	7.9	8	19.1	178	8.1
IDU	51	2.4	3	7.1	54	2.5
MSM	1812	84.1	28	66.7	1840	83.8
MSM & IDU	60	2.8	1	2.4	61	2.8
Other	24	1.1	1	2.4	25	1.1
missing	28	1.3	1	2.4	29	1.3
Prior AIDS-defining condition						
No	1777	82.5	38	90.5	1815	82.6
Yes	378	17.5	4	9.5	382	17.4
Geographical location of enrolling clinic						
Metropolitan	1777	82.5	21	50.0	1798	81.8
Non-metropolitan	378	17.5	21	50.0	399	19.2
Antiretroviral therapy at enrolment						
Naïve	562	26.1	14	33.3	576	26.2
Prior, not current, therapy	395	18.3	2	4.8	397	18.1
Current mono/dual therapy	76	3.5	1	2.4	77	3.5
3+ II,+/−NRTI,+/−NNRTI,+/−PI	16	0.7	0	0.0	100	4.6
3+ NNRTI + PI,+/−NRTI, No II	96	4.5	1	2.4	485	22.1
3+ NRTI + NNRTI, No PI, No II	545	25.3	13	31.0	562	25.6
3+ NRTI + PI, No NNRTI, No II	426	19.8	7	16.7	433	19.7
3+ NRTI, No PI, No NNRTI, No II	39	1.8	4	9.5	43	2.0
CD4 count at enrolment (cells per mm^3^)						
<200	199	9.2	3	7.1	202	9.2
200-299	214	9.9	6	14.3	220	10.0
300-499	650	30.2	13	31.0	663	30.2
500+	989	45.9	18	42.9	1007	45.8
Missing	103	4.8	2	4.8	105	4.8
HIV viral load at enrolment (copies/ml))						
≤400	1245	57.8	25	59.5	1270	57.8
401-10,000	339	15.7	5	11.9	344	15.7
>10,000	470	21.8	10	23.8	480	21.8
Missing	101	4.7	2	4.8	103	4.7

*NRTI* Nucleoside or nucleotide reverse transcriptase inhibitor, *PI* Protease inhibitor, *NNRTI* Non-nucleoside reverse transcriptase inhibitor, *II* Integrase inhibitor

At enrolment, the median age of Indigenous participants was younger than non-Indigenous participants (37 years, IQR 32–46 years vs. 41 years, IQR 35–49 years; *p* = 0.061) but median time since HIV diagnosis was similar (5.0 years, IQR 1.7-9.3 years vs 6.5 years, IQR 2.7-11.8 years; *p* = 0.136). Mean CD4 counts were also similar between Indigenous and non-Indigenous participants at enrolment to AHOD (530/mm^3^(SD 306/mm^3^) & 522/mm^3^(SD 278/mm^3^; respectively; *p* = 0.858). Compared with non-Indigenous participants, a higher proportion of Indigenous participants were female (*p* = 0.029), living in non-metropolitan areas (*p* < 0.001), and reported potential HIV exposures of heterosexual contact (*p* = 0.022) and injecting drug use (*p* = 0.082) (Table 1). In addition, fewer Indigenous participants reported male-to-male sexual contact alone as their most likely HIV risk exposure (*p* = 0.002; Table 1). Most participants, irrespective of Indigenous status, had an undetectable HIV viral load, and over 40 % of both groups had a CD4 count of >500/mm^3^. Similar proportions of Indigenous and non-Indigenous participants were currently infected with hepatitis B (HBsAg positive: 2.4 % vs 3.9 % respectively; *p* = 0.575) or had evidence of current or past hepatitis C infection (HCV antibody positive: 16.7 % vs 10.7 % respectively; *p* = 0.215). There was little difference at enrolment between Indigenous and non-Indigenous participants in total cholesterol (median 4.7 mmol/L vs. 5.0 mmol/L, respectively, *p* = 0.679), HDL (median 1.3 mmol/L vs. 1.1 mmol/L, respectively, *p* = 0.382), triglycerides (median 1.6 mmol/L vs. 1.7 mmol/L, respectively, *p* = 0.620), and blood glucose (median 4.7 mmol/L vs 5.1 mmol/L, respectively, *p* = 0.195) measurements.

Follow-up amongst Indigenous and non-Indigenous participants was 332 & 16270 person-years, respectively. Patient data were available for only 17 (40.5 %) Indigenous and 882 (40.9 %) non-Indigenous participants at the time of commencing their first cART regimen. At initiation of therapy, mean CD4 count (360/mm^3^ vs 430/mm^3^,*p* = 0.301) and proportion starting cART at a CD4 count <200/mm^3^ (26.7 % vs 17.2 %,* p* = 0.335) were similar between Indigenous and non-Indigenous participants, respectively. At the time of cART initiation, few Indigenous (4.2 %) and non-Indigenous (8.8 %) participants had suffered a prior AIDS-defining illness (*p* = 0.715). There was no significant difference between Indigenous and non-Indigenous participants in 1st-line cART regimen prescribed (*p* = 0.879) with most Indigenous (58.3 %) and non-Indigenous (53.1 %) participants being prescribed a non-nucleoside reverse transcriptase inhibitor (NNRTI)-containing regimen.

HIV viral load measurements were available at 12 and 24 months after cART initiation for 20 (47.6 %) and 21 (50.0 %) Indigenous participants and 950 (44.1 %) and 1005 (44.6 %) non-Indigenous participants. Virological suppression (defined as HIV viral load <400 copies/ml) was achieved in similar proportions of Indigenous and non-Indigenous participants at 12 months post-cART initiation (60.0 % vs 71.4 %, *p* = 0.841) and at 24 months post-cART initiation (81.0 % vs 76.5 %, *p* = 0.635).

Indigenous status was not independently associated with shorter time to change from 1st -line to 2nd-line cART regimen (*p* = 0.957) when controlling for factors associated with more rapid change from 1st –line cART regimen (Table 2).

**Table 2:** Multivariate Cox regression analyses for time to change from first- to second- line combination antiretroviral therapy (cART)

	n (%) changed	Time at risk (PY)	Rate of change/100PY	AHR	95 % CI	*p*-value	Overall *p*
Indigenous							0.957
No	416 (37.1)	4946.7	14.2	1	-	-	
Yes	6 (25.0)	112.6	16.0	1.01	0.63-1.63	0.957	
Year of HIV diagnosis							0.662
<1997	148 (27.9)	2429.9	15.8	1	-	-	
≥1997	240 (46.5)	2229.0	12.4	0.96	0.80-1.15	0.662	
Missing	34 (35.0)	400.4	15.7	1.15	0.88-1.52	0.300	
Year of starting cART							<0.001
Prior to 2000	104 (18.5)	2634.5	17.4	1	-	-	
2000 or later	318 (54.6)	2424.8	10.9	0.54	0.46-0.63	<0.001	
Clinic setting							<0.001^#^
General practice	157 (44.5)	1965.9	10.0	1	-	-	
Hospital outpatient	92 (36.8)	958.1	16.5	1.36	1.10-1.69	0.005	
Sexual Health Clinic	173 (32.0)	2135.3	17.2	1.53	1.28-1.83	<0.001	
Prior mono or dual antiretroviral therapy							0.081
No	354 (41.1)	4012.5	12.6	1	1	-	
Yes	68 (24.0)	1046.8	20.5	1.24	0.97-1.59	0.081	
Hepatitis C antibody positive							0.053
No	389 (37.9)	4600.7	13.9	1	-	-	
Yes	33 (28.0)	458.6	18.5	1.25	1.00-1.58	0.053	
Prior AIDS-defining illness							0.304
No	391 (37.4)	4711.4	13.9	1			
Yes	31 (31.3)	348.0	19.5	1.16	0.88-1.52	0.304	
CD4 count at time of commencing cART (per mm^3^)							0.011*
<200	45 (32.1)	531.0	17.9	1	-	-	
200-349	82 (43.9)	808.4	13.0	0.85	0.64-1.13	0.267	
>350	215 (44.7)	2404.0	11.1	0.73	0.57-0.93	0.010	
missing	80 (23.8)	1315.8	19.5	0.96	0.76-1.22	0.746	
HIV viral load at time of commencing cART (copies/ml)							0.200*
≤400	150 (49.3)	1439.0	10.7	1	-	-	
400-10000	124 (41.1)	1426.6	12.5	1.09	0.87-1.36	0.436	
>10000	70 (34.0)	897.2	15.2	1.17	0.92-1.48	0.200	
missing	78 (23.5)	1296.5	19.6	1.22	0.60-2.49	0.582	
First-line cART regimen							<0.001^#^
PI-containing (no NNRTI or II)	104 (23.8)	1535.4	21.7	1	-	-	
NNRTI-containing (No PI or II)	290 (47.6)	3185.3	10.0	0.56	0.47-0.65	<0.001	
Other (± NNRTI ± PI ± II)	28 (28.6)	338.6	20.7	1.01	0.78-1.32	0.927	

Indigenous status included *a priori* in multivariate model; change defined as addition of ≥ 2 new drugs or ≥ 1 new class in regimen, off treatment periods were included in duration of time to change; missing categories not included in calculations of *p*-values. Factors not associated on univariate analysis (*p* > 0.1) with shorter time to change from 1st to 2nd line cART were: gender, age at 1st cART, sexual orientation and past/current Hepatitis B infection

*PY* Person-years, *AHR* Adjusted hazard ratio, *CI* Confidence interval, *cART* combination antiretroviral therapy (at least 3 antiretroviral agents used in combination), *PI* protease inhibitor, *NNRTI* Non-nucleoside reverse transcriptase inhibitor, *II* Integrase inhibitor

* *p* for trend, ^#^ *p* for heterogeneity

The rate of laboratory tests during follow-up by Indigenous status are outlined in Table 3. Indigenous participants were tested significantly less often for HIV markers (CD4 count and HIV viral load), while testing rates of lipids and blood glucose among Indigenous participants were almost half that of non-Indigenous participants.

**Table 3:** Rate of laboratory tests during follow-up by Indigenous status

	Non-Indigenous^a^	Indigenous^b^	*p*-value
Variable	Rate/year (95 % CI)	Rate/year (95 % CI)	
Cholesterol	0.71 (0.70-0.72)	0.43 (0.37-0.51)	<0.001
HDL	0.53 (0.52-0.55)	0.23 (0.18-0.29)	<0.001
Triglycerides	0.71 (0.70-0.72)	0.43 (0.37-0.51)	<0.001
Glucose	0.71 (0.70-0.73)	0.43 (0.37-0.51)	<0.001
CD4 count	2.97 (2.95-3.00)	2.76 (2.58-2.94)	0.025
HIV viral load	2.93 (2.90-2.96)	2.53 (2.36-2.71)	<0.001

^a^non-Indigenous time at risk: 16270 person-years; ^b^Indigenous time at risk: 332 person-years

The rate of death among Indigenous participants during the study period was 0.3/100 person-years (95 % CI 0.07-16.78) compared with 0.93/100 person-years (95 % CI 0.79-2.00) among non-Indigenous participants (logrank *p* = 0.232). There was only one death among Indigenous participants which was attributed to a non-HIV-related condition, while non-HIV causes of death accounted for approximately two-thirds (*n* = 97, 63.8 %) of deaths in non-Indigenous participants.

Loss to follow-up (LTFU) during the study period (defined as no clinic visit for ≥ 12 months) was similar in both groups, occurring among 8 (19.1 %) Indigenous participants (rate 2.41/100 person-years, 95 % CI 1.04-4.75) and 493 (22.9 %) of non-Indigenous participants (rate 3.03/100 person-years, 95 % CI 2.77-3.31) (logrank *p* = 0.531).

## Discussion

As far as we are aware, this is the first study comparing a broad range of HIV clinical outcomes between Indigenous with non-Indigenous Australians. Adverse clinical outcomes appeared no more common among Indigenous participants, although laboratory monitoring of HIV immunological and virological markers, as well as fasting blood glucose and lipids occurred significantly less often among Indigenous participants.

Although numbers of Indigenous participants were relatively small, limiting ability for comparison between groups, the 42 Indigenous participants in fact represent over 10 % of the total number of Indigenous Australians who have ever been diagnosed with HIV [1, 11]. As has been the case with Australian national surveillance of HIV [4, 12], within AHOD there has been a lack of uniform and systematic reporting of Indigenous status. Thus, some data on Indigenous status was missing and 7 of the 27 AHOD study sites do not report Indigenous status for any of their participants, potentially limiting generalisability of our findings. Nonetheless, enrolment characteristics among AHOD participants were broadly similar to characteristics of all Indigenous Australians newly diagnosed with HIV in the last decade, including HIV being diagnosed at younger age, a higher proportion of infections attributed to injecting drug use and smaller proportion attributed to MSM contact [1, 13].

Virological suppression is currently considered the best measure of successful response to cART. Overall in AHOD more than 80 % of treated participants are virologically suppressed in any calendar year over the last decade [14], which is broadly similar to the rates of suppression with various cART regimens in other settings [15–17]. The 2-year virological suppression rates after commencing cART in our substudy participants of around 80 % irrespective of Indigenous status is encouraging, and is likely to reflect good engagement in care.

The significantly lower rate of testing for immunological, virological and other health parameters among Indigenous participants is of concern. The reasons for less frequent laboratory monitoring of Indigenous participants are unclear as such data are not captured in AHOD. However possible reasons could include economic or geographic challenges in accessing care. If such challenges exist for rural and remote Indigenous people living with HIV, formal programs providing social, financial and travel support to enhance HIV monitoring and care may be beneficial in maintaining their long-term health. Missed follow-up appointments may be another possible reason for less frequent monitoring. Missed appointments for HIV care has been shown to be a strong predictor of subsequent virologic failure among overseas clinic populations [18], including among other populations similarly dispossessed as Australia’s Indigenous population [19]. The largest disparity by Indigenous status was observed for frequency of blood glucose and lipid measurements which is perhaps of even greater concern due to the sizeable burden of diabetes and cardiovascular disease among Indigenous Australians [7].

Clinical outcomes appear no worse among Indigenous participants enrolled in AHOD compared with their non-Indigenous counterparts. This may not be representative of the situation among Indigenous patients outside the study. It is likely, at least in part, to reflect engagement in HIV care, as enrolment in AHOD requires attendance at a clinical service providing HIV care. Nonetheless, other health outcomes, such as those among pregnant HIV-positive Indigenous women, have been shown to be equivalent to those among non-Indigenous patients [20], provided that comprehensive, culturally appropriate, multidisciplinary care is provided.

Reasons for LTFU are not collected in AHOD, and could possibly include patients transferring care to another (non-AHOD) clinic or to an AHOD clinic which does not report Indigenous status. We have recently shown higher viral load, longer time under follow-up and prior episodes of LTFU are independent predictors of LFTU in AHOD, although there was no association of LTFU with death [21]. Of note, loss to follow-up among participants in this AHOD substudy, irrespective of Indigenous status, was slightly lower than that among the entire AHOD cohort over the past 15 years (4.04/100PY, 95 % CI 3.73-4.36) [22]. This suggests better engagement with HIV care among participants enrolled at sites reporting Indigenous status, and again may limit generalisablity of our findings to the wider Indigenous HIV-positive population. Adherence is known to be one of the most important predictors of successful virological suppression among HIV-positive individuals [23] which we were unable to assess in this substudy as very few AHOD sites report adherence to cART. Poor adherence can select for drug resistance and can shorten the regimen’s durability [24]. Nonetheless, we examined time to change from 1st to 2nd line therapy as a proxy for adherence and/or tolerability of the initial cART regimen and this was no different by Indigenous status. A further limitation was missing data for a large proportion of participants at time of commencing their first cART regimen and from the 2 years following cART commencement. These clinical markers were mostly unavailable as a result of participants who were recruited to AHOD some years after starting cART, and often at a different clinical service than that at which they were first prescribed cART.

## Conclusion

Indigenous patients in our large clinical cohort had lower rates of laboratory testing for HIV and lipid/glucose parameters than non-Indigenous participants. Overall, lipid and glucose testing among Indigenous participants occurred at under half the frequency that is recommended in national guidelines [25]. Given the elevated risk of cardiovascular disease in the general Indigenous community, the additional cardiovascular risk factor of chronic HIV infection [26] warrants further focus on modifiable risk factors to maximise life expectancy in this population.
